# Effect of Composition Characteristics on Mechanical Properties of UHPMC Based on Response Surface Methodology and Acoustic Emission Monitoring

**DOI:** 10.3390/ma17112714

**Published:** 2024-06-03

**Authors:** Ranran Chen, Yubo Jiao, Mingqi Xiao, Hua Yang, Caiqin Wang

**Affiliations:** 1 Key Laboratory of Urban Security and Disaster Engineering of Ministry of Education, State Key Laboratory of Bridge Engineering Safety and Resilience, Beijing University of Technology, Beijing 100124, China; chen1817159202@163.com (R.C.); yanghua1@emails.bjut.edu.cn (H.Y.);; 2 Shanxi Transportation Technology Research & Development Co., Ltd., Taiyuan 030006, China

**Keywords:** UHPC, manufactured sand, mechanical and flexural properties, response surface methodology, AE monitoring

## Abstract

Manufactured sand (MS) is a promising alternative aggregate to quartz sand (QS) in ultra-high-performance concrete (UHPC) in the preparation of ultra-high-performance manufactured sand concrete (UHPMC), which possesses the characteristics of high strength, low cost, and environmental friendliness. In this study, the effects of variable compositional characteristics including the water–binder ratio, the stone powder (SP) content, and the MS replacement ratio on the mechanical and flexural strength of UHPMC were compared and analyzed based on response surface methodology (RSM). Meanwhile, the damage characteristics of UHPMC during compressive and flexural stress were monitored and evaluated using acoustic emission (AE) technology. The results reveal that the compressive and flexural strengths of UHPMC are both negatively correlated with the water–binder ratio, while they are positively correlated with the MS replacement rate. They tend to firstly increase and subsequently decrease with the increase in the stone powder content. In the load–displacement curve of concrete with a high MS replacement ratio and a low water–binder ratio, the slope in the elastic stage is steeper, the stiffness is higher, and the bending toughness and ductility are also better. The specimens with a 10% to 0% stone powder content present a steeper elastic phase slope, a slightly higher stiffness, and superior ductility. The specimens with a low MS replacement ratio and a high water–binder ratio display earlier cracking and weaker resistance, and the destruction process is complex and very unstable. The damage mode analysis based on RA-AF shows that an increase in the MS replacement ratio and a decrease in the water–binder ratio can both reduce the tensile cracking of UHPMC specimens under a four-point bending test. Although 10% stone powder can marginally slow down crack growth, the failure mode is not significantly affected.

## 1. Introduction

Ultra-high-performance concrete (UHPC) is a novel type of cementitious material with excellent mechanical properties and extremely high durability [1,2,3]. Large-scale projects and specialized structures in numerous nations, including bridges, building elements, nuclear power plants, and coastal engineering, have successfully utilized UHPC [4,5,6]. However, because of its high cost and issues with environmental pollution, the use of UHPC has been restricted in large-scale normal production [7].

An increasing number of scholars are focusing on researching environmentally friendly materials used in the preparation of UHPC. Examples include using crumb rubber [8,9,10] and recycled concrete [11] as substitutes for certain fine aggregates and utilizing fly ash to partially replace cement [12], among other methods. These efforts aim to reduce costs, protect the environment, and simultaneously enhance the mechanical properties of the materials. Natural quartz ore is mined and crushed to create quartz sand (QS), a widely used aggregate in UHPC. In addition to depleting mineral resources, excessive use of natural aggregates threatens the ecology in an irreparable way. Additionally, producing QS via this approach is exceedingly time-consuming and expensive, and it can seriously pollute the environment with silica dust [13]. Therefore, finding ecologically benign and resource-efficient aggregates to replace QS in UHPC production has become crucial.

Manufactured sand (MS) is a type of artificial sand with a particle size of less than 4.75 mm which is created from rock fragments (excluding soft and worn rocks) after mining, mechanical crushing and screening [14]. The raw materials used for MS ranges from natural raw materials to waste resources such as tailings and waste rocks. For instance, using granite and other stone products produces a lot of waste [15,16,17,18,19,20,21]. The local manufacturing of MS can lower production and transportation costs. As a fine aggregate in concrete, MS has been utilized increasingly frequently [22]. Shen et al. [23] made UHPC with a standard strength utilizing gradation optimization, demonstrated the viability of using MS as the aggregate instead of QS for preparing UHPC. Chu et al. [24] explored the feasibility of preparing green UHPC with MS and discovered that the green UHPMC possessed excellent mechanical properties. Felekoglu [25] investigated the effects of different stone powder contents on the workability of limestone-based MS concrete. Their results indicated that when the stone powder content rises, mixed concrete’s water requirement rises as well. Currently, numerous researchers have established the viability of substituting MS for QS and investigated the impact of single factors on UHPMC performance, such as the MS replacement rate, the stone powder content, and the fiber content. Nonetheless, the current research remains severely limited, and little is known about the working performance and mechanical properties of UHPMC under the influence of multi-factor coupling.

The destruction of UHPC materials is a process in which microscopic damage accumulates over time. The study of damage mechanisms plays an important role in further studying UHPC materials’ properties. The non-destructive testing (NDT) approach has emerged as the primary method for analyzing the fracture and deformation process of UHPC under various stress conditions. Commonly used NDT methods include X-ray assessment [26], ultrasonic assessment [27], acoustic emission (AE) technology [28], and digital image correlation (DIC). AE technology is presently employed extensively in the damage detection and evaluation of concrete and masonry bridges [29]. Akichika et al. [30] accurately characterized the damage extent of reinforced concrete beams using the cumulative energy dissipation index of acoustic emissions. Bai et al. [31] used AE technology to reveal the concrete deterioration mechanism at various temperatures through ringing counts and *b*-values. Xu et al. [32] investigated the fracture behavior of rubberized steel fiber (SF) recycled aggregate concrete using AE parameters based on the rise angle (RA) and average frequency (AF). Zhang et al. [33] demonstrated the feasibility of using information entropy analysis to track the initial cracking process of concrete shear walls. At present, numerous results have been achieved regarding the damage detection of concrete materials or components using AE parameters. Therefore, AE technology can more effectively monitor the fracture process of UHPMC beams.

In this study, a novel environmentally friendly UHPMC was prepared by replacing QS in conventional UHPC with MS. The effects of the water–binder ratio (0.16, 0.18, 0.2), MS replacement ratio (20%, 60%, 100%), and stone powder (SP) content 0%, 5%, 10%) on the compressive strength, flexural strength of beams, and flexural toughness of UHPMC were investigated by means of compressive strength tests and four-point bending tests. AE technology was applied to monitor the fracture process of UHPMC compressive and four-point bending tests simultaneously. The effects of different material factors on the working and mechanical properties of UHPMC were analyzed based on RSM. The fracture characteristics, fracture processes and fracture modes of UHPMC beams under different material conditions were analyzed using AE energy, ringing counts, information entropy, *b*-value and RA-AF.

## 2. Materials and Methods

### 2.1. Raw Materials

**Figure 1:**
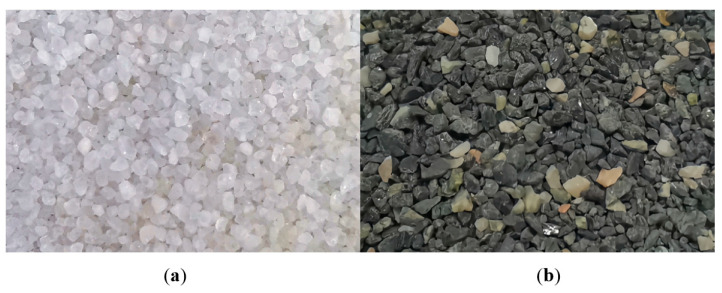
Aggregate appearance: (**a**) QS; (**b**) MS.

**Figure 2:**
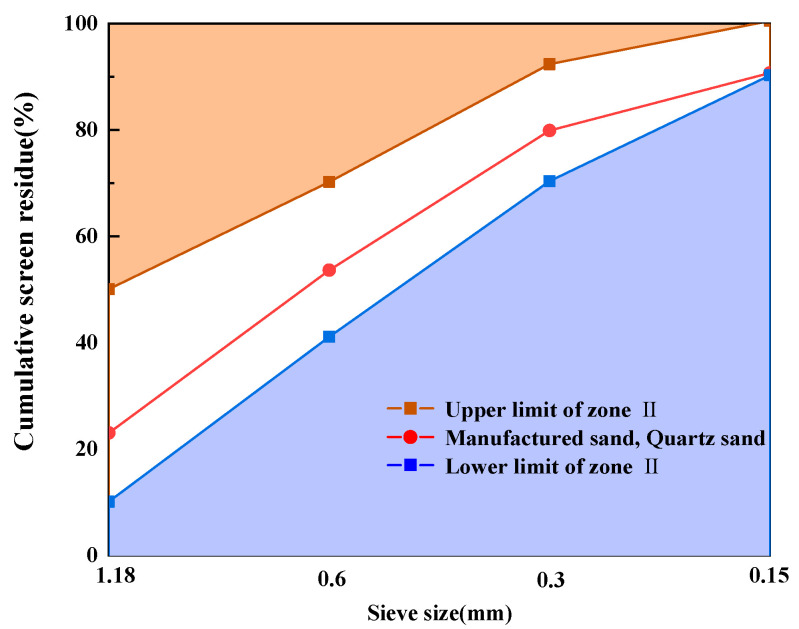
Gradation curve of MS and QS.

The appearance of the MS is illustrated in Figure 1, and this MS was produced through the wet process in Sanhe City, Hebei Province, China. Through the screening test, MS and QS were found to have the same particle size distribution curve, as shown in Figure 2. The main performance indicators of MS and QS are shown in Table 1. Other raw materials and specific properties are described in detail in [34].

**Figure 3:**
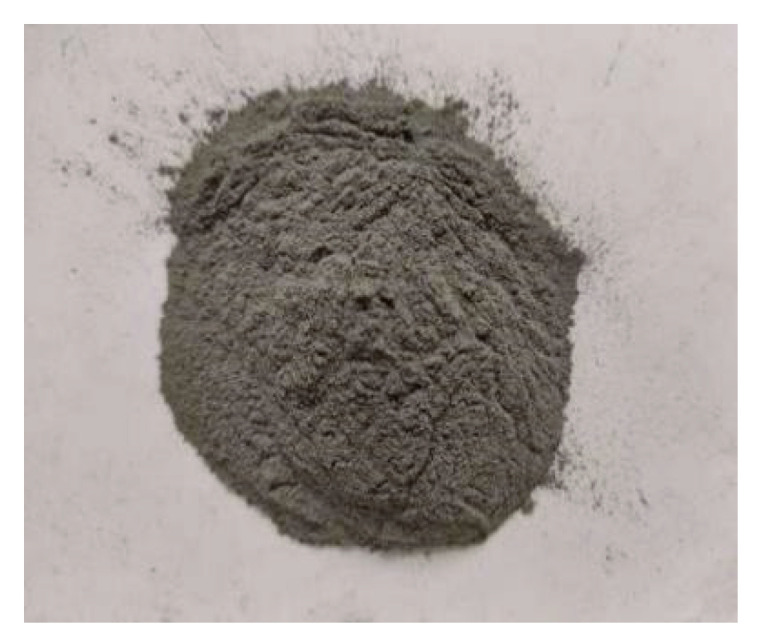
Appearance of stone powder.

The stone powder used in this test is the raw material of the MS described above, which is ground and screened with a ball mill. Its particle size is less than 0.075 mm. The appearance of stone powder is shown in Figure 3.

### 2.2. Proportion of Mixes

Based on RSM, QS was replaced by MS with different replacement rates (20%, 60%, 100%), MS stone powder (SP) contents (0%, 5%, 10%), and water–binder ratios (0.16, 0.18, 0.20) by using the equal-mass substitution method to design the mix ratio.

The UHPC mix used in this study mainly consisted of P.O 52.5 ordinary silicate cement, silica fume (SF), quartz powder (QP) with an average particle size of 15 μm, QS, 13 mm steel fibers, and high-performance polycarboxylic acid water-reducing additive (HPWRA). The UHPMC mix ratio design for this study is shown in Table 2. 

### 2.3. Specimen Preparation

For each mix ratio, UHPMC specimens with the dimensions 100 mm × 100 mm × 100 mm and 100 mm × 100 mm × 400 mm were designed and fabricated in this study. In each group, three parallel specimens were prepared for testing, and the corresponding average was considered representative. During fabrication, to make the specimen structure uniform and avoid internal particle agglomeration, the UHPC raw materials were added to the mixer and then all mixed according to the following procedure: (1) we poured cement, SF, aggregate, QP and other dry components into the mixer according to the mixing ratios listed in Table 1. (2) After 3 min of low-speed mixing in the mixer, we added water and the water reducer. Within two minutes, we added the water reducer gradually after diluting it by half. We gradually added the remaining water and water reducing agent during the next 2 min mixing process. (3) Steel fibers were slowly added and mixed for another 8 min to prevent the agglomeration of the fibers. (4) Finally, the mixes were demolded and allowed to cure for 28 days in a standard curing environment at 20 °C and 95% RH.

### 2.4. Test Procedure

#### 2.4.1. Mechanical Test

**Figure 4:**
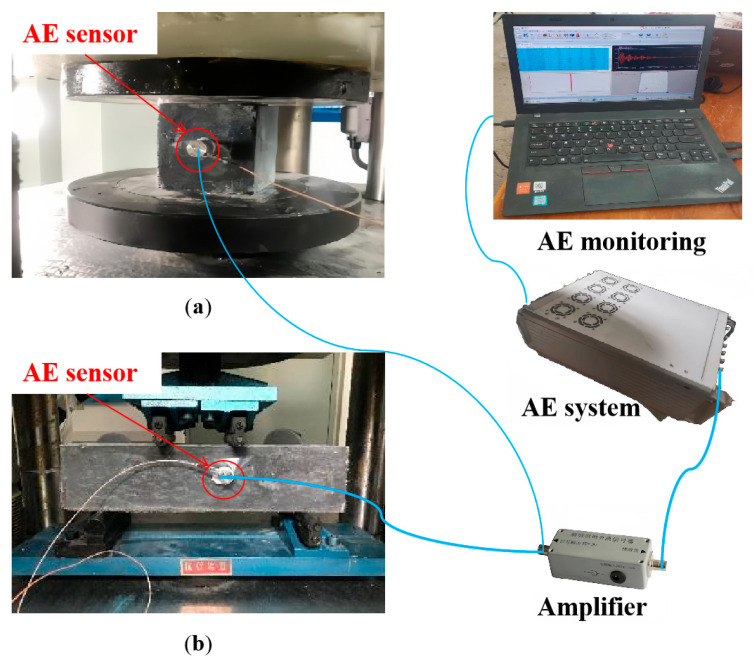
Mechanical and AE test system diagram: (**a**) compressive test and (**b**) four-point bending test.

Compressive strength tests and four-point bending tests were carried out according to GB/T50081-2019 [35]. The compressive strength of the concrete specimens was tested on a 2000 kN universal testing machine (UTM) at a loading rate of 0.8 MPa/s. The four-point bending tests were conducted using a 300 kN UTM under a loading rate of 0.04 MPa/s. Figure 4 illustrates the mechanical and AE test system diagram.

#### 2.4.2. AE Test

To characterize the damage process of concrete under different loading conditions, AE monitoring was simultaneously conducted with mechanical tests. The twelve-channel data acquisition system was adopted as shown in Figure 4, and the sensor was firmly adhered to the side of each specimen through a coupling agent. The threshold of the AE acquisition system was set to 45 dB and the acquisition frequency was set to 5 MSPS.

**Figure 5:**
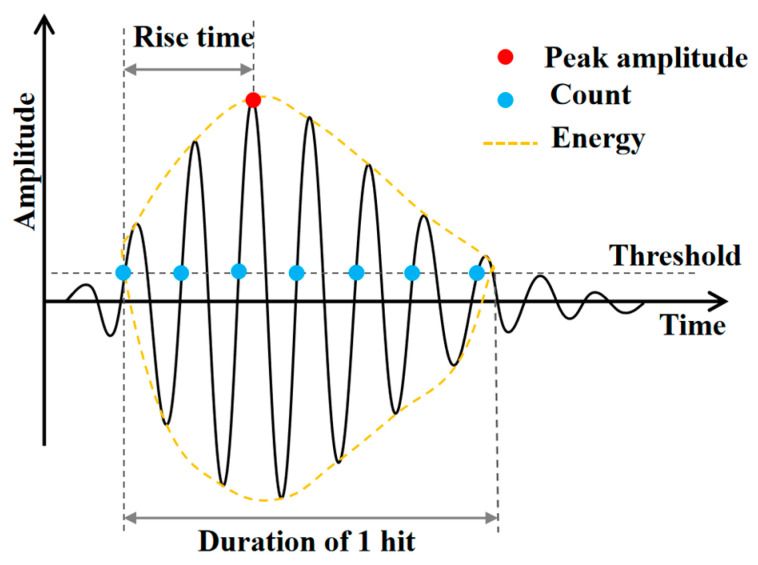
Typical AE waveform and AE parameters [36].

In this study, AE parameters such as ringing count, energy, information entropy, *b*-value and RA-AF were used to compare the fracture characteristics of UHPMC under different material conditions. Typical AE signals and key parameters are shown in Figure 5 [36].

#### 2.4.3. Parameter Index

1. Flexural toughness

Flexural toughness is an important indicator of the mechanical properties of steel fiber concrete. The flexural toughness of UHPMC beams under different material factors was expressed as the flexural toughness ratio *R*, which was calculated according to JG/T 470-2015 [37].

The flexural toughness of the specimen before the peak load corresponding to the deflection *δ_p_* is characterized by the initial flexural toughness ratio *R_e,p_*, which be obtained according to Equations (1) and (2):

(1) $$R_{e,p}=\frac{f_{e,p}}{f_{ftm}}$$

(2) $$f_{e,p}=\frac{\Omega_{p}L}{bh^{2}\delta_{p}}$$

where *R_e,p_* is the initial flexural toughness ratio; *f_e,p_* is the equivalent initial flexural strength, MPa; *δ_p_* is the mid-span deflection at peak load, mm; Ω*_p_* is the area under the load–deflection curve for a mid-span deflection of *δ_p_*, N∙mm; and fftm is the flexural strength of the steel fiber concrete, MPa.

2. Displacement ductility ratio

The displacement ductility ratio [38], which is the ratio of ultimate displacement to yield displacement, describes the inelastic deformation capability of a component without significantly degrading the initial front and rear bearing capacity. The calculation formula is represented by Equation (3) [39].

(3) $$\mu =\frac{\Delta p}{\Delta y}$$

where *µ* is the ductility index; Δ*p* is the midspan displacement at the peak load; and Δ*y* is the midspan displacement at the yielding load.

3. AE parameter

AE ringing count is the number of ringing pulses collected that exceed a threshold. AE energy is the elastic energy released from the internal damage of the test beam, reflecting the intensity of the signal. AE ringing counts and energies are positively correlated with crack development in concrete and are suitable for the qualitative analysis of damage development in concrete.

The *b*-value is an indicator of incipient damage. A stable *b*-value indicates that the concrete is mainly in a micro-cracked state, while a decrease in the *b*-value indicates the development of micro-cracks in concrete. The formula for the *b*-value is represented by Equation (4) [40,41].

(4) $$\mathrm{lg}N=a-b\left(\frac{A_{dB}}{20}\right)$$

where *b* is the *b*-value, which indicates the relationship between the magnitude and the number of events; *A*_d*B*_ is the peak amplitude of AE hit; *N* is the cumulative number of AE events with an amplitude greater than *A*_d*B*_/20; and *a* is an empirical constant.

Information entropy can be used to indicate the degree of disorder of AE energy. When the energy system is ordered, the information entropy is at a relatively low value; when the energy system is more disordered, the information entropy is at a relatively high value [42]. The mathematical expression for information entropy is presented in Equation (5).

(5) $$H(x)=-\sum_{i=1}^{n}p_{AE}^{i}\log p_{AE}^{i}$$

where *H*(*x*) is the information entropy; *p^i^_AE_* is the *i*th AE energy probability; and *n* = 50, meaning that every 50 consecutive AE energy events are grouped together.

**Figure 6:**
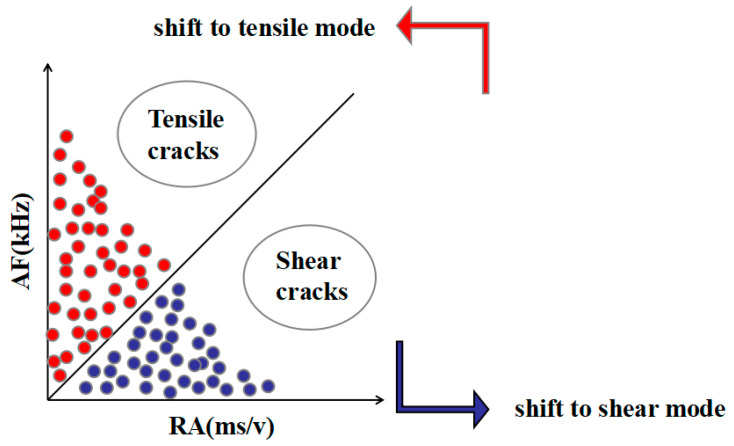
Typical indication diagram of RA and AF [36].

RA is the ratio of rise time to amplitude in AE events, and AF is the ratio of counts to duration in AE events. In the traditional method, the RA-AF dividing line is used to distinguish between tensile failure mode and shear failure mode, as shown in Figure 6. However, it is impossible to determine the exact boundary. The Gaussian mixture model (GMM) is verified to be the most suitable algorithm for the classification of acoustic emission cracks [43,44]. Therefore, GMM was used for crack classification in this paper.

## 3. Results

### 3.1. Mechanical Properties Analysis Based on RSM

Based on RSM, the influence of the three independent variables (MS replacement ratio *X*_1_, stone powder content *X*_2_, and water–binder ratio *X*_3_) on predicting response values (compressive strength *Y*_1_, flexural strength *Y*_2_, and flexural toughness ratio *Y*_3_) was investigated. An equation was established accordingly. The significance of the developed models and model terms was assessed through ANOVA results (*R*_2_, Adj. *R*_2_, *p*-value, *F*-value) at a 95% confidence level. *p*-values below 0.05 indicate that the model and variables are significant for the response values.

#### 3.1.1. Compressive Strength

Calculations showed that the model for the compressive strength significant variable (*p* < 0.05) comprises *X*_1_, *X*_3_, and *X*_22_. After removing non-significant variables, the linear compressive strength model based on coding factors is obtained:

(6) $$Y_{1}=121.9+2.69X_{1}-4.79X_{3}-4.31X_{22}$$

**Figure 7:**
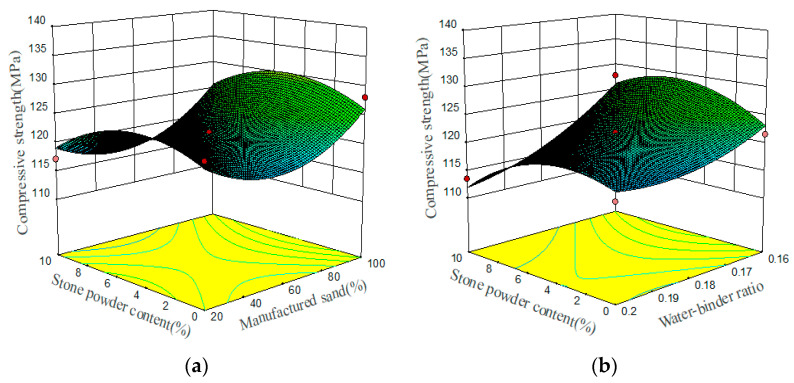
Three-dimensional response surface plots between preparation parameters and compressive strength: (**a**) stone powder content and MS replacement ratio; (**b**) water–binder ratio and stone powder content (The red points represent design points where the actual values are higher or lower than the predicted values).

The compressive strength prediction model demonstrates that three main factors influence the flexural toughness ratio, ranked in order of impact: the water–binder ratio, the stone powder content, and the MS replacement ratio. The relationships between these three preparation parameters and the compressive strength are demonstrated in the 3D response surface plots, which are shown in Figure 7. The effect of the water–binder ratio on the compressive strength is the most significant. The compressive strength increases as the water–binder ratio decreases. With the increase in the stone powder content, compressive strength shows a tendency to increase and then decrease. The reason for this is that the appropriate amount of stone powder can fill the aggregate gap, so that the UHPMC is more compact, which leads to an increase in compressive strength. However, stone powder does not participate in the hydration reaction, and excessive stone powder will weaken the strength of the cement paste, lowering its compressive strength. The compressive strength also increases when the MS replacement ratio increases, but the effect is insignificant. The main reason for this is that the surface of MS has many edges and high roughness, which increases the friction between UHPMC aggregates and provides a certain biting force, thereby enhancing the compressive strength of concrete.

#### 3.1.2. Flexural Strength

The calculation results indicate that the models with significant variables (*p* < 0.05) for bending strength *Y*_2_ comprise *X*_1_, *X*_3_, *X*_22_. After removing non-significant variables, the linear compressive strength model based on coding factors is obtained:

(7) $$Y_{2}=13.29+0.57X_{1}-0.96X_{3}-1.29X_{22}$$

**Figure 8:**
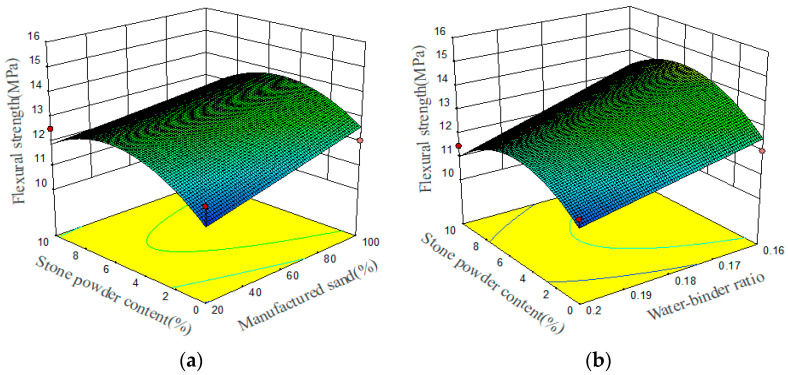
Three-dimensional response surface plots between preparation parameters and flexural strength: (**a**) stone powder content and MS replacement ratio; (**b**) water–binder ratio and stone powder content (The red points represent design points where the actual values are higher or lower than the predicted values).

The flexural strength prediction model identifies the significance levels of the three independent variables, ranked in order of impact: the water–binder ratio, the MS replacement ratio, and the stone powder content. The relationships between these three preparation parameters and flexural strength are demonstrated in the 3D response surface plots, which are shown in Figure 8. The changes in the bending and compressive strengths of UHPMC are consistent. As the water–binder ratio decreases, the flexural strength increases. The flexural strength shows a trend of first increasing and then decreasing with the increase in stone powder content. As MS replacement ratio increases, the flexural strength also increases. Similarly, the influence mechanism of UHPMC bending strength is the same as that of compressive strength.

#### 3.1.3. Flexural Toughness

The calculation results indicate that the models with significant variables (*p* < 0.05) for bending toughness ratio comprise *X*_1_, *X*_11_, and *X*_33_. After removing non-significant variables, the linear flexural toughness ratio model based on coding factors is obtained:

(8) $$Y_{3}=0.55+0.031X_{1}-0.097X_{11}-0.10X_{33}$$

**Figure 9:**
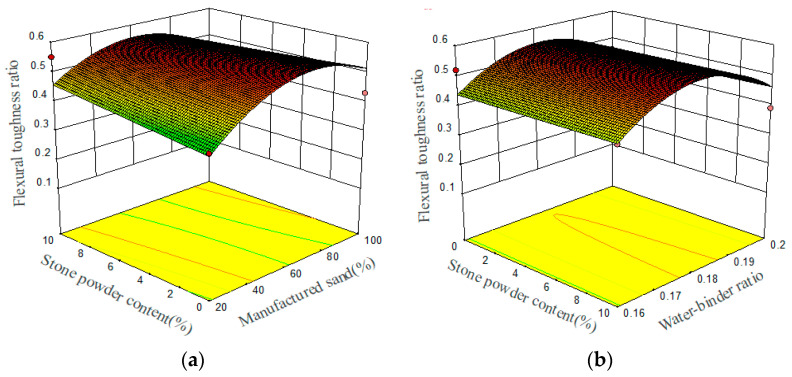
Three-dimensional response surface plots between preparation parameters and flexural toughness ratio: (**a**) stone powder content and MS replacement ratio; (**b**) water–binder ratio and stone powder content (The red points represent design points where the actual values are higher or lower than the predicted values; The differently colored curves at the bottom are projection graphs of the three-dimensional surface, reflecting the same meaning as the three-dimensional surface itself).

The flexural strength prediction model underscores the limited influence of these three key factors on the flexural toughness ratio, namely the microsilica (MS) replacement ratio, the water–binder ratio, and the stone powder content, in descending order of significance. The bending toughness of steel fiber concrete is mainly manifested by the initial flexural toughness ratio, and the higher the *R_e_* and *p*-values, the better the toughness. The relationships between these three preparation parameters and the flexural toughness ratio are demonstrated in the 3D response surface plots, which are shown in Figure 9. As the water–binder ratio decreases, the flexural toughness ratio decreases, and as the MS replacement ratio increases, the toughness ratio also increases. The stone powder content has a negligible effect on the flexural toughness ratio. The flexural toughness of steel fiber concrete is mainly influenced by the amount of fiber added. Since the same amount of steel fiber was used throughout the experiment, there was no significant change in the flexural toughness ratio. Therefore, replacing QS with MS will have a positive impact on the flexural toughness of UHPMC beams.

#### 3.1.4. Combinatorial Optimization Forecasting

Based on the above analysis, the optimal combination prediction of the three-factor ratio was established. We established the ideal goals for compressive strength, bending strength and flexural toughness ratio. The optimal solution of the three-factor ratio was predicted as shown in Table 3.

### 3.2. Load–Displacement Curve

#### 3.2.1. Load–Displacement Curve of Compression Tests

The failure process of UHPMC was examined in conjunction with the load–displacement curve and the compressive test phenomenon. From initial loading to specimen failure, the UHPMC compressive specimen goes through four stages: the contact compaction stage, the elastic stage, the crack generation and development stage, and the failure stage. During the contact compaction stage, the displacement increases rapidly. The characteristics of the elastic stage include a sharp increase in load with small displacement increments, a steep slope of the load–displacement curve, and the generation of some small cracks inside the UHPMC. In the stage of crack development, the load continues to rise, and the UHPMC specimen eventually reaches its maximum compressive strength. The internal microcracks in the specimen grow quickly into larger ones, and sound is generated during the internal crack development of the specimen, indicating that the specimen is about to fail. The characteristics of the failure stage include the rapid development and penetration of internal cracks in UHPMC specimens, forming macroscopic large cracks, and the appearance of many vertical cracks and peeling loss of bearing capacity on the surface of the specimens.

1. Water–binder ratio

**Figure 10:**
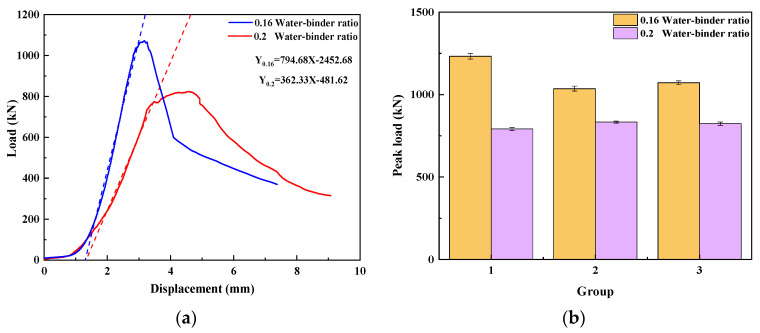
Water–binder ratio: (**a**) load–displacement curve; (**b**) peak load in compression (The dashed line represents the slope of the load-displacement curve in the elastic stage).

The compressive strength load–displacement curves of UHPMC with a water–binder ratio of 0.16 and 0.2 are shown in Figure 10a. The peak load of the three groups of 0.16 and 0.2 water–binder ratio specimens is shown in Figure 10b. As can be seen, the compressive strength of UHPMC specimens with a water–binder ratio of 0.16 is significantly higher when controlling the MS replacement ratio and stone powder content to be the same. By fitting the tangent line of the elastic stage, it is found that the slope of the curve (794.68) at a water–binder ratio of 0.16 is steeper than the slope of the curve at a water–binder ratio of 0.2 (362.33), and the failure stage of the 0.16 water–binder ratio specimen sharply decreases.

The amount of cement in the low-water–binder ratio specimen is increased. The hydration reaction leads to the increased production of cementitious material products, thereby enhancing the inter-aggregate bonding within the test specimens. It can be concluded that as the water–binder ratio decreases, the strength and stiffness of the UHPMC specimen increase.

2. MS replacement ratio

**Figure 11:**
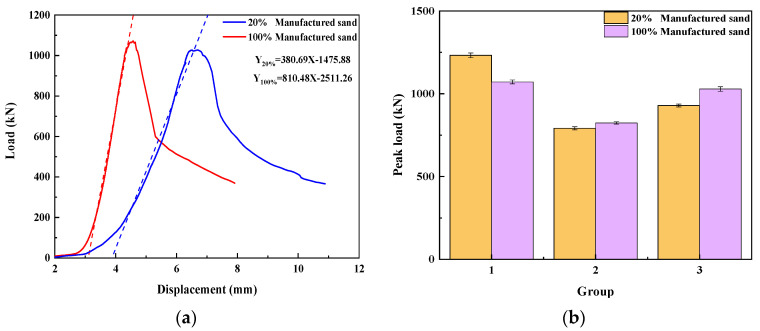
MS replacement ratio: (**a**) load–displacement curve; (**b**) peak load in compression.

The UHPMC load–displacement curves with a 20% and 100% MS replacement ratio are shown in Figure 11a. The peak load of the three sets of 20% and 100% MS replacement ratio specimens is shown in Figure 11b. When the water–binder ratio and stone powder content are controlled to be the same, an increase in MS replacement ratio can improve the compressive strength of UHPMC. It can be seen that the 20% MS group experiences larger displacement during the elastic stage, indicating that the cracks and deformation displayed by the 20% MS replacement ratio group are greater than those displayed by the 100% MS replacement ratio group during the elastic stage. By fitting the tangent of the elastic stage, it can be seen that the slope of the 100% MS replacement ratio group (810.48) is steeper than the slope of the 20% MS replacement ratio group (380.70).

The mechanical bonding force between aggregates and between aggregates and cementitious materials and the bonding strength between interfaces can all be significantly improved by MS particles, thus increasing the compressive strength of UHPMC. As can be seen, the strength of the UHPMC specimen and the stiffness both improve as the MS replacement ratio increases.

3. Stone powder content

**Figure 12:**
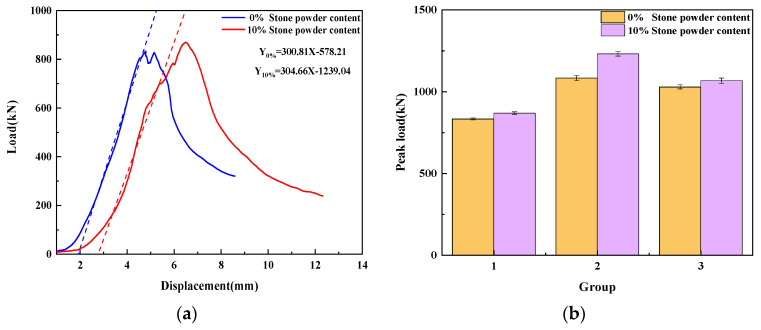
Stone powder content: (**a**) load–displacement curve; (**b**) peak load in compression.

The UHPMC load–displacement curves with a 0% and 10% stone powder content are shown in Figure 12a. The peak load of the three sets of 0% and 10% stone powder content specimens is shown in Figure 12b. As can be observed, the compressive strength of the specimen with a 10% stone powder content is marginally higher than that of the specimen with a 0% stone powder content when the water–binder ratio and MS replacement ratio are both equal. The 10% stone powder content specimen has a greater displacement in the elastic stage, indicating that its deformation is large in the elastic stage and its resistance is weak. It can be observed that there is not much of a difference between the slopes of the curves for the specimens with 0% and 10% stone powder contents (300.81 and 304.66, respectively) by fitting the tangent of the elastic stage of the load–displacement curve.

Stone powder can fill the gaps between aggregates, making UHPMC more compact and increasing compressive strength. However, the specimens with a 10% stone powder content have a higher percentage of stone powder, which lowers the fraction of coarse particles, weakening the skeletal effect. Therefore, the 10% stone powder content specimens have a somewhat higher stiffness than the 0% stone powder content specimens.

#### 3.2.2. Load–Displacement Curves of Four–Point Bending Tests

The UHPMC beam undergoes four stages from initial loading to failure, including the contact compaction stage, the elastic stage, the crack generation and development stage, and the failure stage. In this study, we combine the load–displacement curves to analyze the specimens’ ductility. The yield displacement was calculated using the isoenergetic method, as shown in Table 4.

1. Water–binder ratio

**Figure 13:**
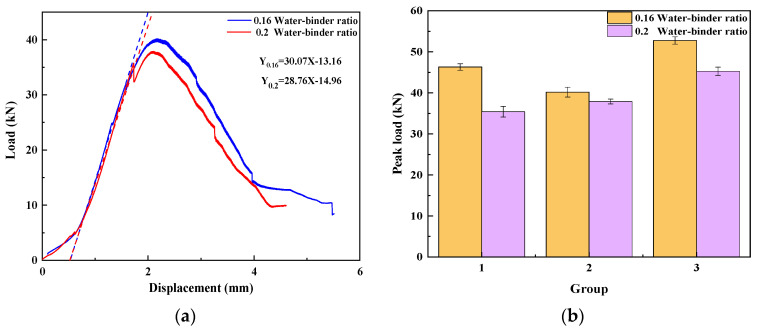
Water–binder ratio: (**a**) load–displacement curve; (**b**) peak load of four–point bending tests (The dashed line represents the slope of the load–displacement curve in the elastic stage).

Figure 13a illustrates the flexural strength load–displacement curves of UHPMCs with water–binder ratios of 0.16 and 0.2. Figure 13b shows the peak load of beams with a water–binder ratio of 0.16 and 0.2. Notably, UHPMC beams exhibit significantly higher flexural strength at a water–binder ratio of 0.16, accompanied by a slightly greater displacement ductility ratio. By fitting the tangent of the elastic stage, it is evident that the slope of the curve for a water–binder ratio of 0.16 (30.8885) surpasses that of the curve for a water–biner ratio of 0.2 (15.1126), and the UHPMC strength increases with the decrease in the water–binder ratio. This indicates that a decrease in the water–binder ratio enhances the stiffness and ductility of UHPMC during the elastic stage.

2. MS replacement ratio

**Figure 14:**
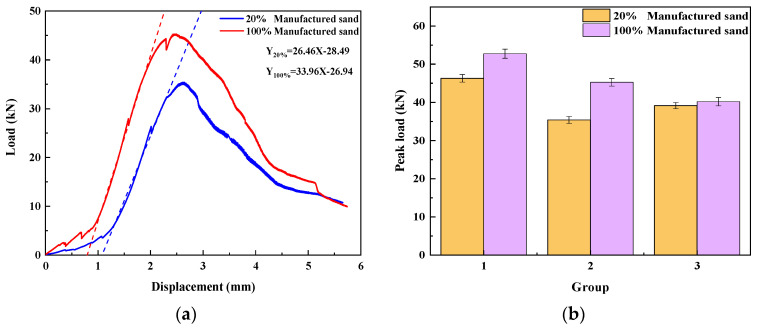
MS replacement ratio: (**a**) load–displacement curve; (**b**) peak load of four–point bending tests (The dashed line in represents the slope of the load–displacement curve in the elastic stage).

Figure 14a displays the UHPMC load–displacement curves with 20% and 100% MS replacement ratios, While Figure 14b illustrates the peak load of these beams. Maintaining a constant water–binder ratio and stone powder content, enhancing the MS replacement ratio enhances the flexural strength of UHPMC and increases displacement ductility. The 20% MS replacement ratio group produces larger cracks and deformations during the elastic stage. Fitting the tangent line during the elastic stage reveals that the load–displacement curve slope of the 100% MS replacement ratio group (21.8593) exceeds that of the 20% MS replacement ratio group (15.3739). As the MS replacement ratio increases, both the stiffness and displacement ductility ratio of UHPMC during the elastic stage experience augmentation.

3. Stone powder content

**Figure 15:**
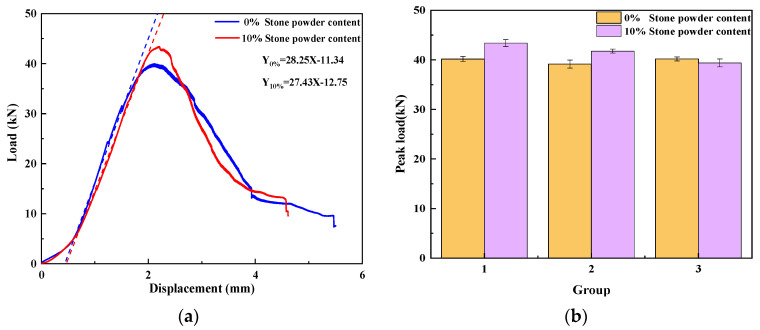
Stone powder content: (**a**) load–displacement curve; (**b**) peak load of four–point bending test (The dashed line in represents the slope of the load–displacement curve in the elastic stage).

The UHPMC load displacement curves with 0% and 10% stone powder content are shown in Figure 15a. The peak load of the three groups of 0% and 10% stone powder content beams is shown in Figure 15b.

It was found that the control water–binder ratio and MS replacement ratio were the same, and the peak bending load of the 10% stone powder content specimens was marginally higher than that of the 0% stone powder content specimens when comparing the peak loads of the three groups. The bending strength is slightly larger. It can be determined that the slope of the curve in the elastic stage is not significantly different by fitting the tangent of the load–displacement curve’s elastic phase. The 10% stone powder content specimen experiences a sharp drop. It is evident that the stiffness and elastic modulus of the 0% specimen are slightly higher and the ductility is better when compared to the 10% stone powder content beam.

### 3.3. Fracture Analysis of UHPMC under Compression Based on AE Parameters

#### 3.3.1. Effect of Water–Binder Ratio

1. AE energy

**Figure 16:**
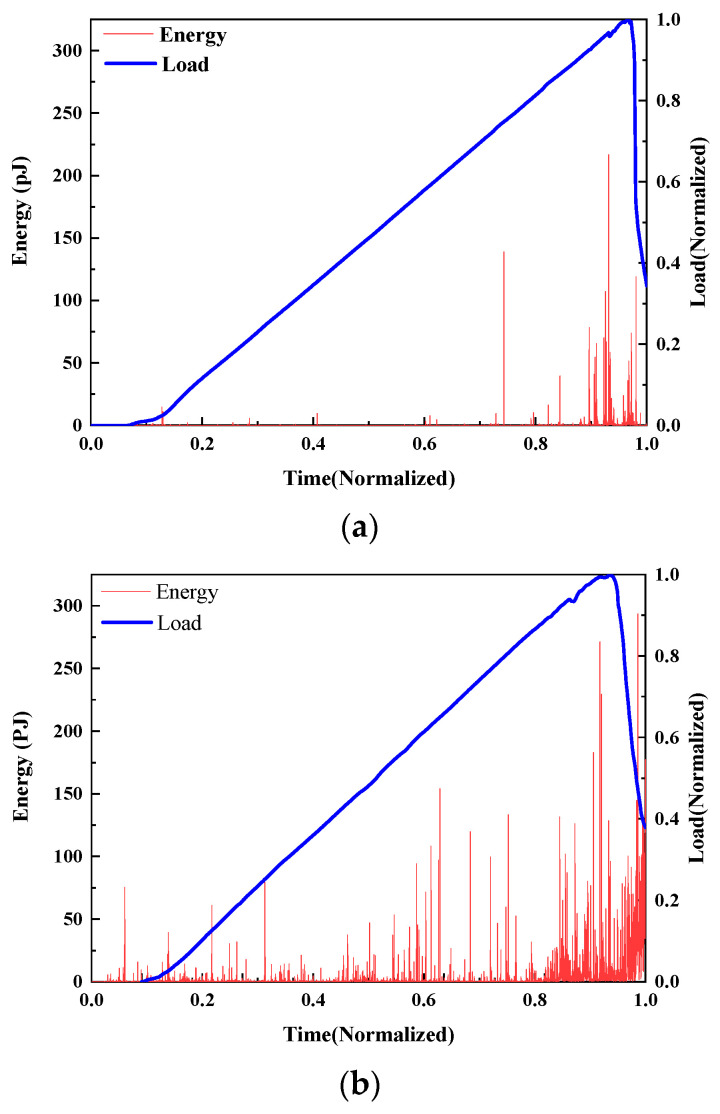
Curves of the load and AE energy of UHPMC with different water–binder ratios: (**a**) 0.16 water–binder ratio; (**b**) 0.2 water–binder ratio.

This study investigates the damage evolution of UHPMC specimens with varying water–binder ratios through the analysis of load curves and AE energy. The representative specimens were selected according to the control variable method. The load curves and AE energy curves of M100-S5-W0.16 and M100-S5-W0.2 specimens with water–binder ratios of 0.16 and 0.2 are shown in Figure 16.

Prior to reaching the 0.7 load level, the specimen with a water–binder ratio of 0.16 is in an elastic stage characterized by minimal fluctuation, with only a sparse occurrence of microcracks inside the specimen. Between the load levels of 0.7 and 0.9, the specimen initiates energy dissipation, accompanied by the development and propagation of microcracks. Subsequently, within the 0.9 to 1.0 load level, a significant release of energy occurs, leading to the penetration of cracks until ultimate specimen failure. Conversely, the energy release and generation of microcracks in the 0.2 water–binder ratio specimen are less pronounced before reaching the 0.5 load level. Beyond the 0.5 load level, with increased loading, a higher magnitude of energy is liberated, and as the cracks penetrate, the energy signal released becomes more concentrated. Notably, the M100-S5-W0.16 specimen generated lower energy throughout the damage process compared to the M100-S5-W0.2 specimen, with the latter demonstrating an earlier onset of crack formation. This discrepancy can be attributed to the enhanced bonding force and compressive strength between aggregates in specimens with lower water–binder ratios, resulting in heightened resistance to deformation and delayed energy absorption and release.

2. *b*-value

**Figure 17:**
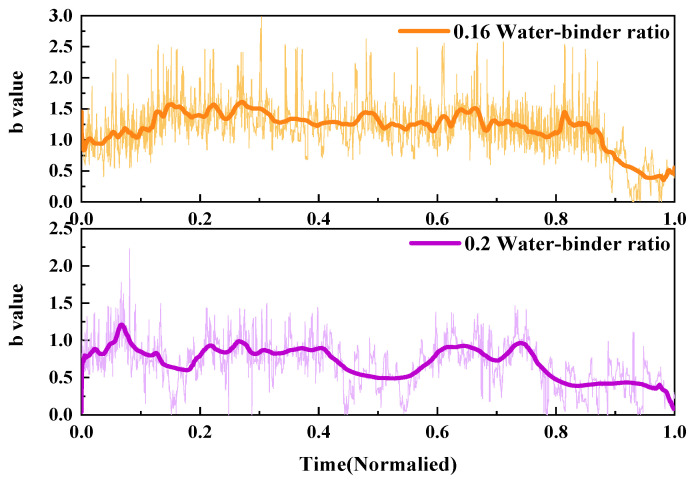
Comparison of the AE *b*-value of UHPMC with different water–binder ratios.

The fracture process of UHPMC specimens with different water–binder ratios was characterized and compared using the AE *b*-value. Similarly, two samples, labeled as M100-S5-W0.16 and M100-S5-W0.2 specimens with water–binder ratios of 0.16 and 0.2, were selected for evaluation. The *b*-value curves are presented in Figure 17.

During the compressive test, the AE *b*-value of concrete specimen exhibits a downward, violently fluctuating, and sharply declining trend. When there are numerous tiny fractures inside the concrete, *b*-value accumulates and increases. As cracks propagate, *b*-value decreases. Macroscopic large cracks develop in the specimen as the damage mounts. When the cracks penetrate and cause a sharp decrease in the *b*-value, the specimen will fail.

The application of average sliding filtering on the *b*-value can enhance the visibility of *b*-value variations and reflect the development of internal cracks in the specimen. The stable fluctuation stage of the M100-S5-W0.16 specimen was comparatively protracted. The M100-S5-W0.16 specimen’s cracks rapidly expanded into macroscopic large cracks with a rapid fall in the *b*-value between the 0.8 and 1.0 time periods. The M100-S5-W0.2 specimen exhibits a higher rate of crack propagation and an earlier final failure time. At a time of 0.5, the *b*-value dramatically dropped, indicating the occurrence of large cracks. After a time of 0.7, the *b*-value sharply declines, forming a through crack. The high water–binder ratio specimen fails earlier during the loading process, resulting in more complicated cracks and an unstable failure process.

#### 3.3.2. Effect of MS Replacement Ratio

1. AE energy

**Figure 18:**
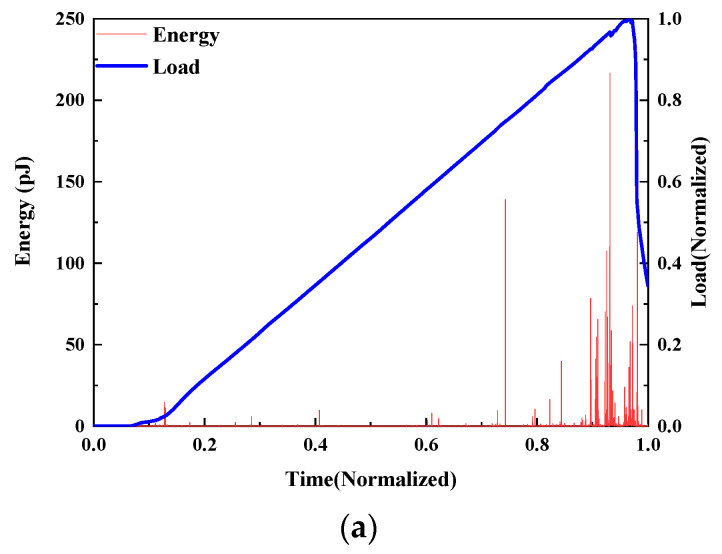

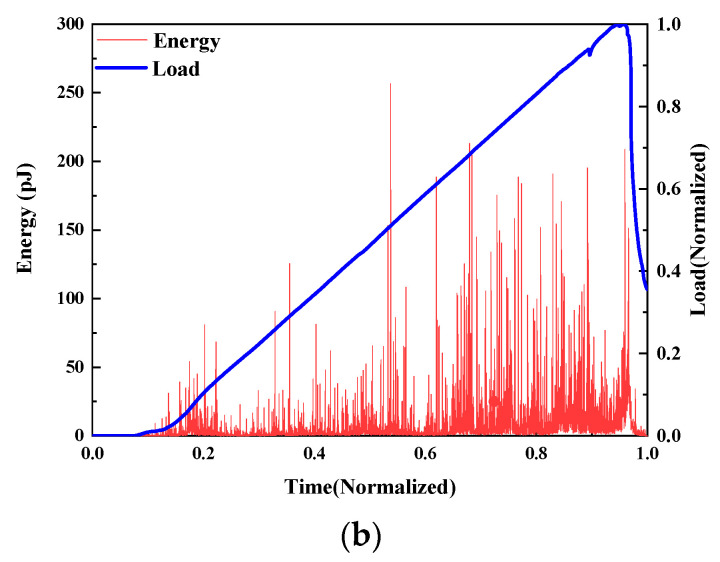
Curves of the load and AE energy of UHPMC with different MS replacement ratios: (**a**) 20% MS replacement ratio; (**b**) 100% MS replacement ratio.

The damage evolution of UHPMC specimens with different MS replacement ratios were characterized and contrasted using load curves and AE energy. According to the control variable, the AE energy of M20-S5-W0.16 and M100-S5-W0.16 with MS replacement ratios of 20% and 100% was selected, as shown in Figure 18.

At the 0.2 load level, the M20-S5-W0.16 specimen releases a significant AE energy signal that caused numerous microcracks inside the specimen. At 0.6 load, significantly more energy is released, which is indicative of the significant interior damage and fracture propagation of specimen. However, the M100-S5-W0.16 specimen shows a strong compressive capability, and no substantial energy signal is generated throughout the 0–0.7 time period. There are no obvious cracks or failures at this point. At a load of 0.7, significant AE energy is released inside the specimen until it is destroyed. The M20-S5-W0.16 specimen cracks earlier and fails more quickly.

2. *b*-value

**Figure 19:**
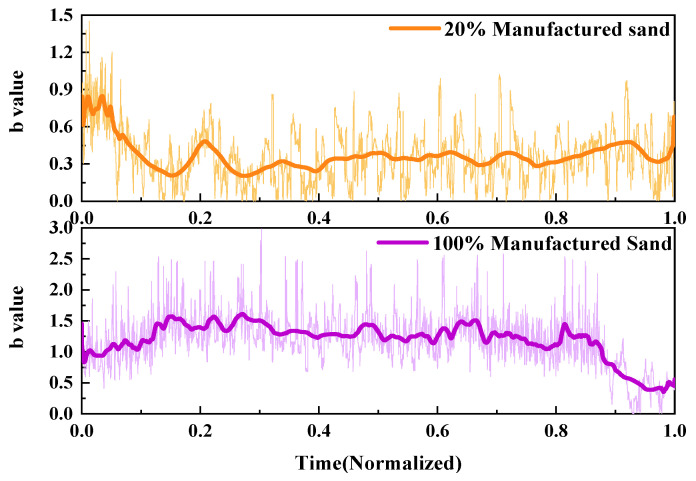
Comparison of the AE *b*-value of UHPMC with different MS replacement ratios.

Using the AE *b*-value, the fracture process of UHPMC specimens with different MS replacement ratios was characterized and compared. Similarly, two sample M20-S5-W0.16 and M100-S5-W0.16 specimens with MS replacement ratios of 20% and 100% were selected. The *b*-value curves are displayed in Figure 19.

The initial AE *b*-value of the M20-S5-W0.16 beam is relatively high, indicating a higher frequency of micro-events during this stage. As the stress gradually increases, a significant decrease in *b*-value is observed at a time of 0.2, indicating the initiation of microcracks in the specimen. Between the 0.3 and 0.8 time periods, the *b*-value exhibits significant fluctuations, indicating rapid crack propagation and foreshadowing the transition of cracks towards failure. The M100-S5-W0.16 beam shows rapid and consecutive decreases in *b*-value after a time of 0.7, indicating rapid and extensive macro-cracking, forming macroscopic cracks until the specimen fails. The variation in *b*-value reflects a relatively late failure of the M100-S5-W0.16 beam, with a more stable crack development in the early stages.

#### 3.3.3. Effect of Stone Powder Content

1. AE energy

**Figure 20:**
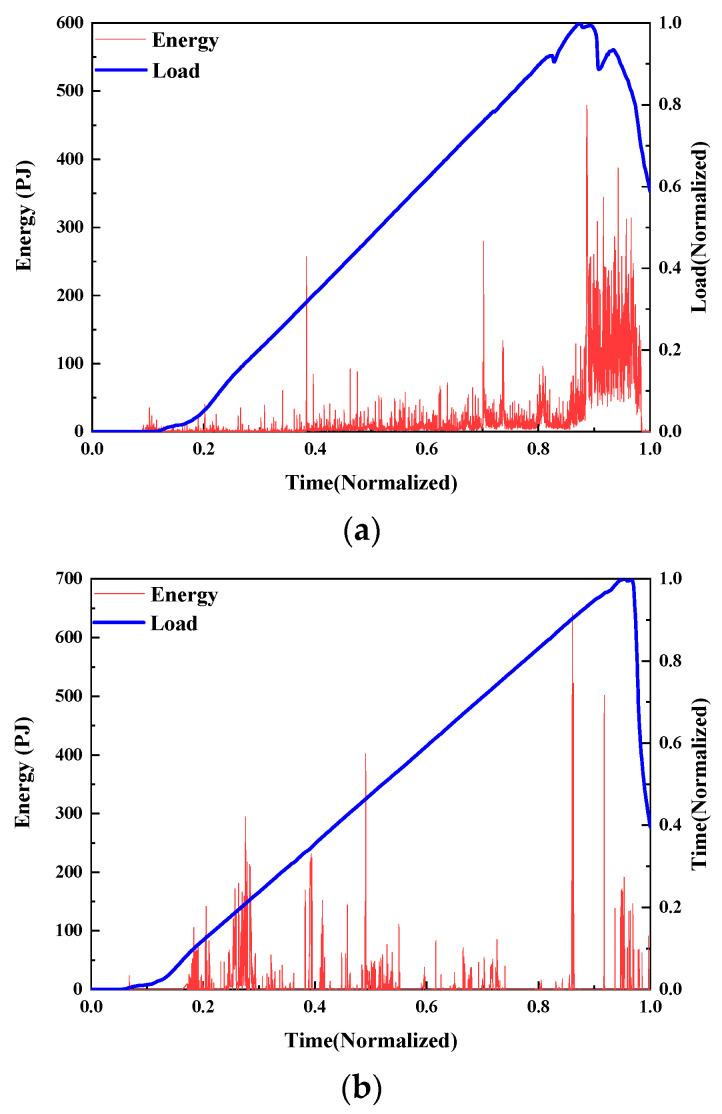
Curves of the load and AE energy of UHPMC with different stone powder contents: (**a**) 0% stone powder content; (**b**) 10% stone powder content.

According to the control variable method, the objects of analysis are the load curves and AE energy of M60-S0-W0.2 and M60-S10-W0.2 specimens with 0% and 10% stone powder content, as shown in Figure 20.

The M60-S0-W0.2 specimen released a significant amount of energy, indicating that huge cracks started to appear at the 0.2 load level. Subsequently, the rapid release of energy indicates that the crack development is relatively rapid. The AE energy of M60-S10-W0.2 specimen is comparatively low prior to the 0.8 load, with only two significant energy releases that both successfully prevent the formation of cracks. Between the 0.8 and 1.0 load level, both specimens experience significant energy release as the load increases, resulting in specimen failure. Among the specimens, M60-S10-W0.2 releases more energy. The 10% stone powder content specimen exhibits a later onset and final failure of cracks than the 0% stone powder content specimen. The primary reason for this is that stone powder can enhance the compactness and deformation resistance of UHPMC.

2. *b*-value

**Figure 21:**
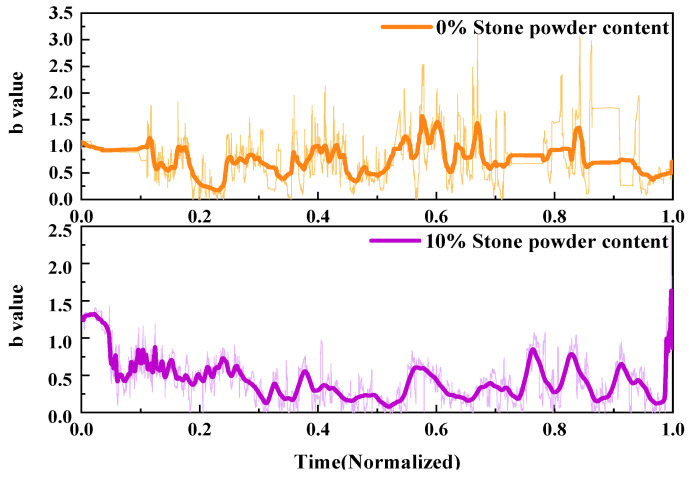
Comparison of the AE *b*-value of UHPMC with different stone powder contents.

Similarly, two samples were selected to characterize fracture process of UHPMC specimens using the AE *b*-value. We used the M60-S0-W0.2 and M60-S10-W0.2 specimens with stone powder contents of 0% and 10%. The *b*-value curves are shown in Figure 21.

As can be observed, both *b*-values exhibit a general tendency to decrease, fluctuate violently, and sharply decrease. At a time of 0.1, a rapid decrease in the *b*-value indicates the occurrence of large cracks, and the M60-S10-W0.2 specimen’s fluctuation amplitude is higher. After a time of 0.1, the *b*-value drastically fluctuates, resulting in the emergence of small cracks and the spread of huge fissures. The overall *b*-value of the M60-S0-W0.2 specimen is relatively large and fluctuates violently, ultimately leading to the formation of macroscopic cracks and failure. The M60-S10-W0.2 specimen’s overall *b*-value variation is comparatively constant, and cracks progress without difficulty until the specimen fails due to peeling. This indicates that the M60-S10-W0.2 specimen has a stronger compressive strength and a more stable failure compared to the M60-S0-W0.2 specimen.

### 3.4. Flexural Damage Analysis of UHPMC under Four–Point Bending via AE Parameters

#### 3.4.1. Effect of the Water–Binder Ratio

1. Ringing count

**Figure 22:**
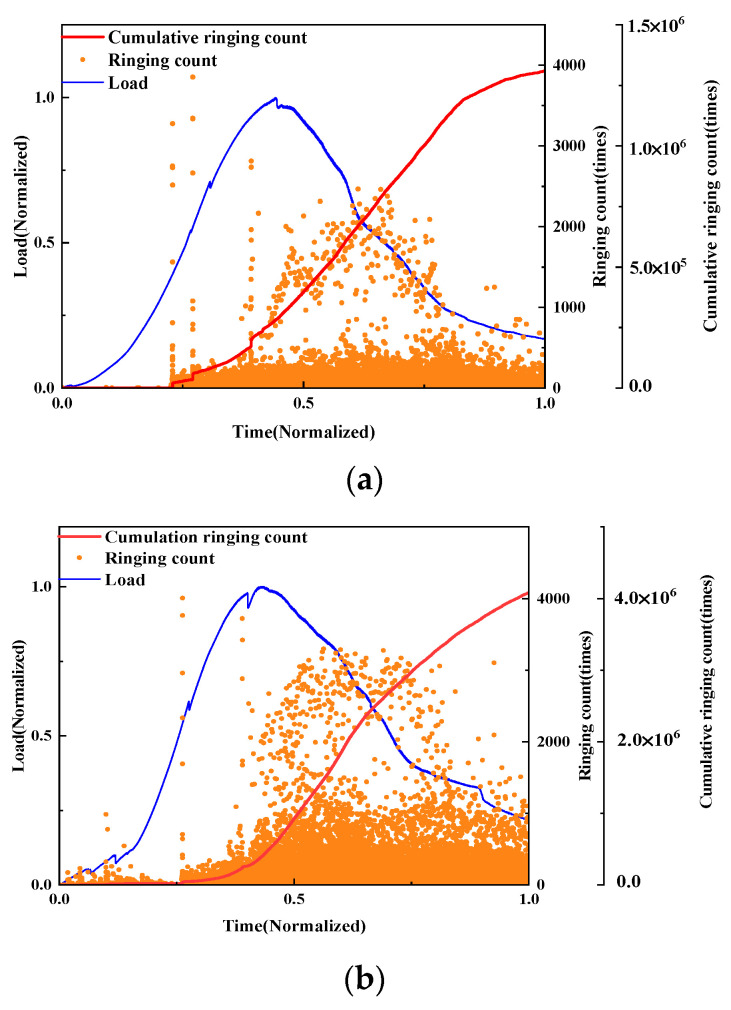
Curves of the load and AE ringing count of UHPMC with different water–binder ratios: (**a**) 0.16 water–binder ratio; (**b**) 0.2 water–binder ratio.

The damage evolution of UHPMC beams with different water–binder ratios were characterized and compared through load curves and AE ringing counts analysis. Employing the control variable method, beams designated as M100-S5-W0.16 and M100-S5-W0.2, corresponding to water–binder ratio of 0.16 and 0.2, respectively, were selected for detailed analysis, as illustrated in Figure 22.

From the graph, it can be observed that the AE ringing counts collected before a 0.5 load for both groups of specimens are not significant, indicating the elastic loading phase. However, for the M100-S5-W0.2 beam, there is a sudden increase in AE ringing counts exceeding 4000 at the 0.5 load level, indicating crack initiation. After reaching the peak load, intense AE ringing counts are present, indicating severe crack generation and propagation. In contrast, the overall AE ringing counts for the M100-S5-W0.16 beam during the entire loading period are relatively low, resulting in cumulative ringing counts below 1.5 × 10^6^. This suggests that the damage process for the M100-S5-W0.16 beam is relatively mild, with a lower degree of damage. Therefore, beams with a higher water–binder ratio exhibit earlier crack initiation, faster crack propagation during loading, and more severe internal damage in the specimen.

2. Information entropy

**Figure 23:**
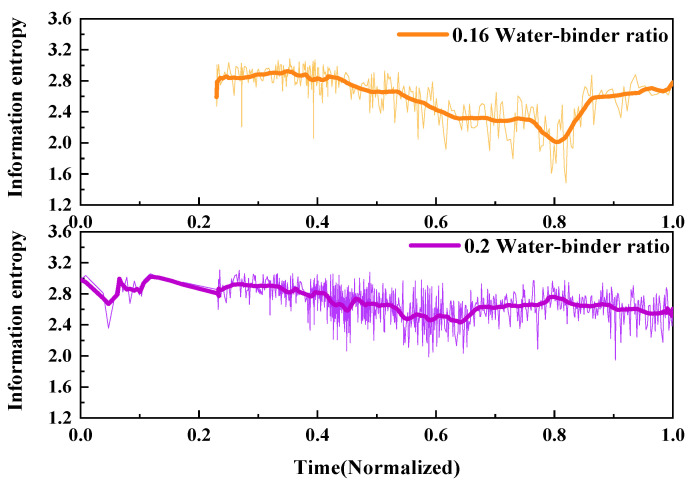
Comparison of the AE information entropy of UHPMC with different water–binder ratios.

The fracture behavior of UHPMC specimens with different water–binder ratios was characterized and contrasted utilizing AE information entropy analysis. The comparative information entropy curves for the M100-S5-W0.16 and M100-S5-W0.2 beams with water–binder ratios of 0.16 and 0.2, respectively, is shown in Figure 23.

During the 0–0.2 time period, the information entropy of both beams remained relatively stable. At this point, the energy values displayed disarray, with only minor fissures appearing within the sample. During the time period from 0.3 to 0.9, both the information entropy fluctuated continuously and decreased, accompanied by the occurrence of alternating large and small cracks, resulting in continuous damage to the sample. After the 0.8 time point, the information entropy continued to fluctuate, while the sample damage tended to stabilize.

The information entropy of the M100-S5-W0.2 beam decreased earlier and fluctuated more frequently compared to M100-S5-W0.16 beam. These findings imply that the specimens with a higher water–binder ratio underwent earlier crack generation and specimen failure during the loading process, and the failure process was more unstable.

3. Fracture mode analysis based on RA and AF

**Figure 24:**
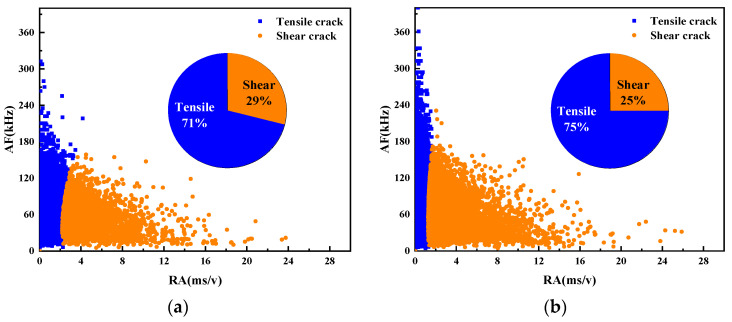
Scatter plots of RA and AF of UHPMC with different water–binder ratios: (**a**) 0.16 water–binder ratio; (**b**) 0.2 water–binder ratio.

The fracture modes of UHPC beams with different water–binder ratios were compared according to the AE parameters RA and AF. Figure 24 illustrates the scatter plot of RA and AF for the beam specimens numbered M100-S5-W0.16 and M100-S5-W0.2, possessing water–binder ratios of 0.16 and 0.2.

The analysis reveals that AE signals predominantly exhibit high AF values and low RA values, indicating that more tensile cracks than shear cracks are generated in the damage process of the specimen. Overall, the failure mechanism of beams under four-point bending primarily stems from tensile stresses. A decrease in the water–binder ratio from 0.16 to 0.2 leads to increased tensile damage and reduced shear damage. The main reason for this is that the low water–binder ratio of the specimen reduces internal pores and makes its structure more dense. Consequently, the bending strength is improved, thereby weakening the tensile signal while enhancing the shear signal.

#### 3.4.2. Effect of the MS Replacement Ratio

1. Ringing count

**Figure 25:**
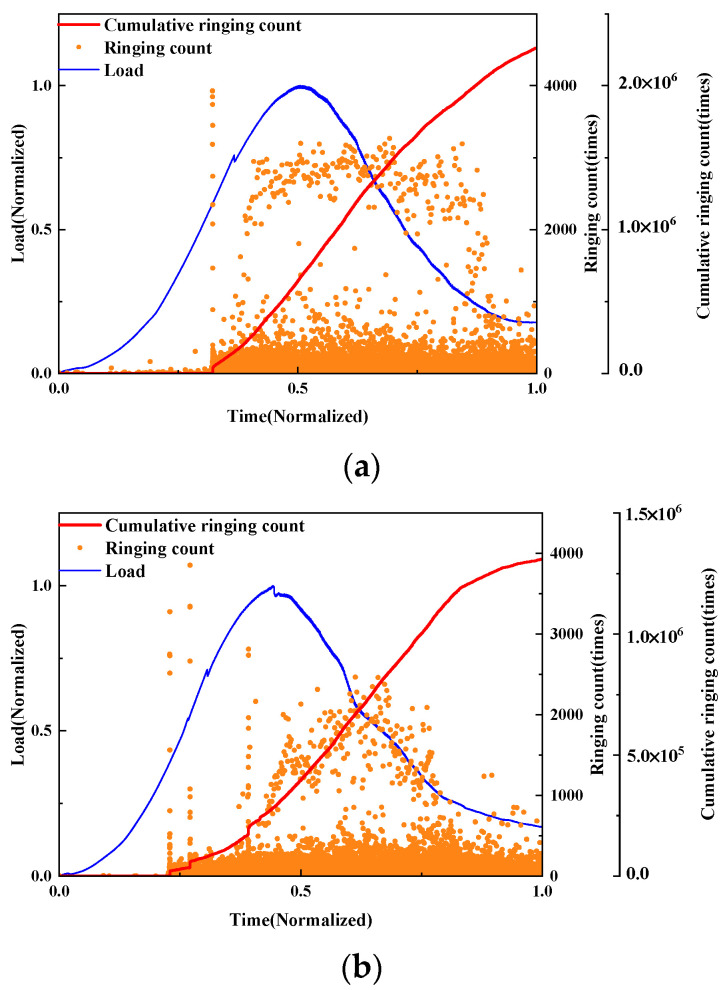
Curves of the load and AE ringing count of UHPMC with different MS replacement ratios: (**a**) 20% MS replacement ratio; (**b**) 100% MS replacement ratio.

The damage evolution of UHPMC specimens with different MS replacement ratios was characterized and compared using load curves and AE ringing counts. The AE ringing counts and load curves of the M20-S5-W0.16 and M100-S5-W0.16 beams with 20% and 100% MS replacement ratio sand are shown in Figure 25.

It can be known that the No. 1 beam has microcracks at the 0.2 load level. At the 0.6 load level, when the ringing count exceeds 4000, the cumulative ringing count suddenly increases. Afterwards, the ringing count is around 3000, and the cumulative ringing count continues to rise, causing cracks to develop quickly. Cracks begin to develop in the M100-S5-W0.16 beam at the 0.4 load level. During the elastic stage, three larger ringing counts are generated, and the cumulative ringing count suddenly increases, indicating the occurrence of larger cracks. After the 0.8 load level, the cumulative ringing count continues to increase and cracks continue to expand. The overall ringing count of the M100-S5-W0.16 beam is lower than that of the M20-S5-W0.16 beam, and the load level during the generation and main failure stages of beam cracks is relatively high, indicating that the M100-S5-W0.16 beam has stable crack propagation and strong resistance to deformation.

2. Information entropy

**Figure 26:**
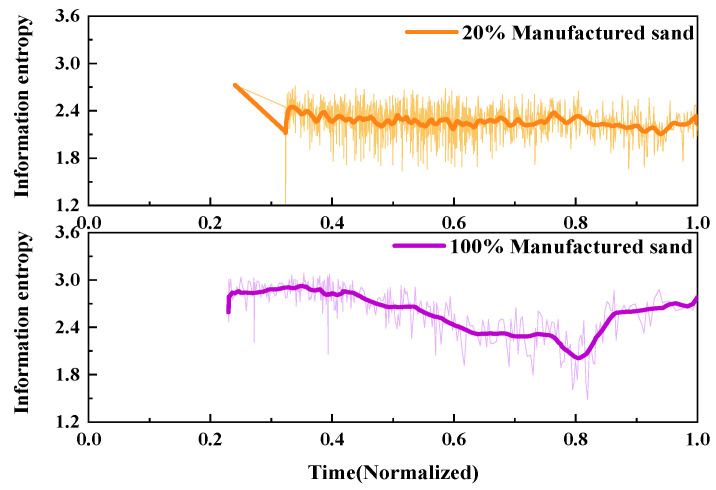
Comparison of AE information entropy of UHPMC with different MS replacement ratios.

The fracture process of UHPMC specimens with different MS replacement ratio was characterized and compared using AE information entropy. The comparison of the information entropy curves of the M20-S5-W0.16 and M100-S5-W0.16 beams with MS replacement ratio of 20% and 100% is shown in Figure 26.

It is evident that the No. 1 beam had significant decreases at a time of 0.3. At this point, large cracks have already been generated. Between the 0.4 and 0.6 time points, the information entropy fluctuates violently, releasing a large amount of energy, indicating that the beam is rapidly failing. After a time of 0.2 for the M100-S5-W0.16 beam, the information entropy continues to fluctuate and decrease, and large and small cracks alternate until the damage progresses gradually. The appearance of significant cracks in No. 1 beam occurs earlier than that in the M100-S5-W0.16 beam, with a more complex failure mode. It can be observed that the higher the MS replacement ratio, the stronger the resistance to deformation in the early stage, and the simpler the failure mode.

3. Fracture mode analysis based on RA and AF

**Figure 27:**
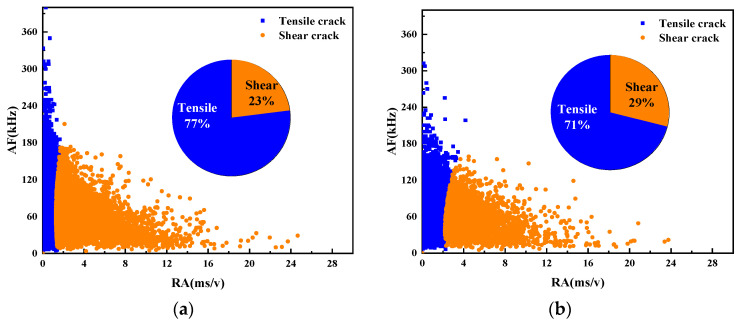
Scatter plots of RA and AF of UHPMC with different MS replacement ratio: (**a**) 20% MS replacement ratio; (**b**) 100% MS replacement ratio.

The fracture modes of UHPC beams with different MS replacement ratios were compared according to the AE parameters RA and AF. The RA and AF scatter plots of the M20-S5-W0.16 and M100-S5-W0.16 beam specimens with MS replacement ratios of 20% and 100% are shown in Figure 27.

The results show that the M100-S5-W0.16 beam experiences less tensile damage than the No. 1 beam, while suffering more shear damage. This trend aligns with the observations from the AE ringing count and information entropy analysis mentioned previously. The increase in the MS replacement ratio strengthens the bonds between aggregates, thereby enhancing their resistance to tensile deformation. Consequently, shear damage increases and tensile damage reduces as the MS replacement ratio increases.

#### 3.4.3. Effect of Stone Powder Content

1. Ringing count

**Figure 28:**
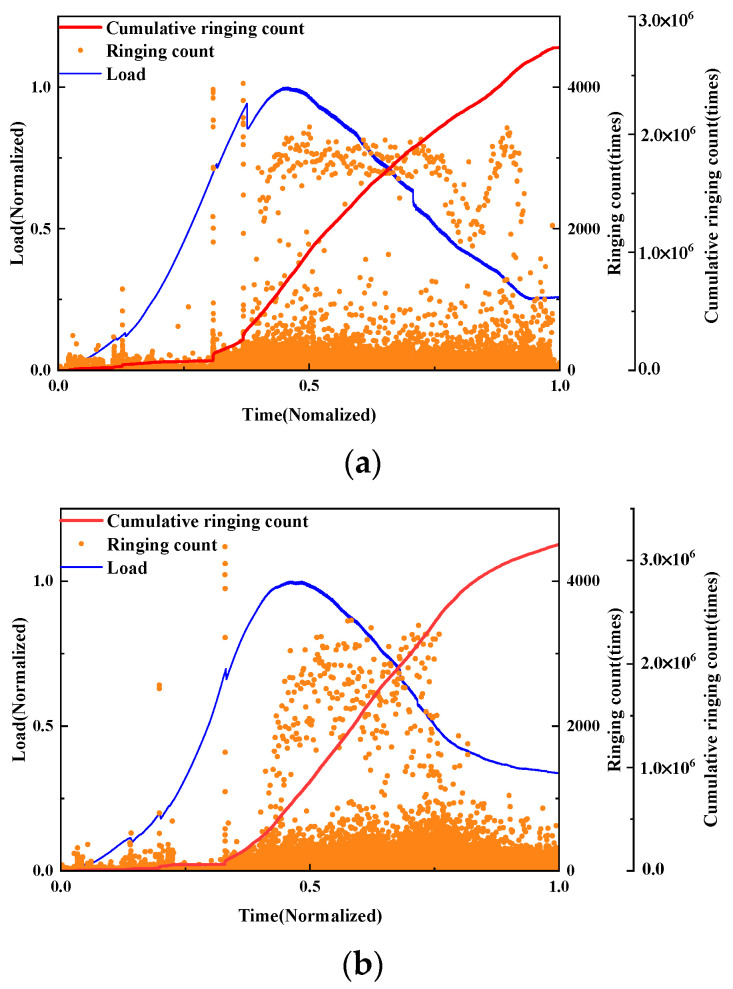
Curves of the load and AE ringing count of UHPMC with different stone powder contents: (**a**) 0% stone powder content; (**b**) 10% stone powder content.

The damage evolution of UHPMC specimens with different stone powder contents was characterized and compared utilizing load curves and AE ringing counts. Employing the control variable method, the AE ringing counts and load curves of the M60-S0-W0.2 and M60-S10-W0.2 beams with 0% and 10% stone powder contents, respectively, are shown in Figure 28.

The destruction process of both beams is basically similar. At the 0.7 load level, significant ringing counts are present in both beams, and a dramatic rise in the cumulative ringing counts indicates the presence of large cracks. Afterwards, frequent high ringing counts are generated, showing the quick development of cracks. As the maximum load is approached, the M60-S10-W0.2 beam exhibits elevated AE ringing counts. High ringing counts appear early and frequently in the M60-S0-W0.2 beam, and the damage is quite severe. It can be observed that beams with a 10% stone powder content have stronger bonds than beams with a 0% stone powder content, and their early resistance is also significantly stronger.

2. Information entropy

**Figure 29:**
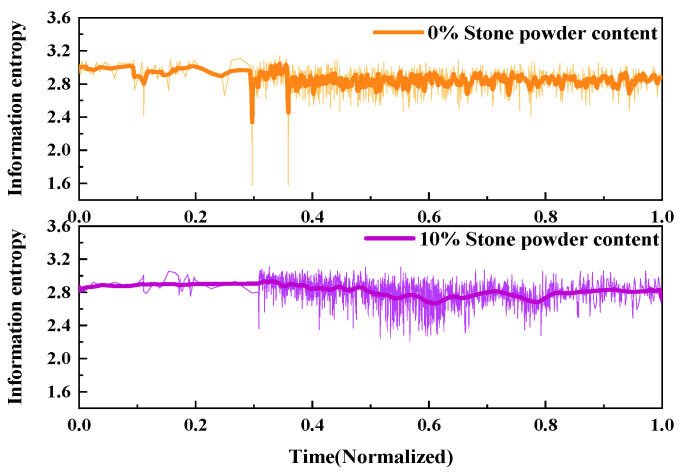
Comparison of the AE information entropy of UHPMC with different stone powder content.

The fracture process of UHPMC specimens with different stone powder contents was characterized and compared using AE information entropy. Figure 29 presents the comparison of information entropy curves for the beams M60-S0-W0.2 and M60-S10-W0.2, representing 0% and 10% stone powder content, respectively.

As can be seen, the information entropy curves of beams with different stone powder contents exhibit three distinct stages: horizontal oscillation, decline, and stable development. The decline section of the M60-S0-W0.2 beam is more significant, with a wider range of decline, indicating the expansion of larger cracks. The overall variation range of the M60-S10-W0.2 beam is relatively minor, indicating the generation of microcracks and the stable propagation of microcracks. This indicates that the M60-S10-W0.2 beam possesses superior uniform load capacity compared to the M60-S0-W0.2 beam, retarding the formation and propagation of large cracks.

3. Fracture mode analysis based on RA and AF

**Figure 30:**
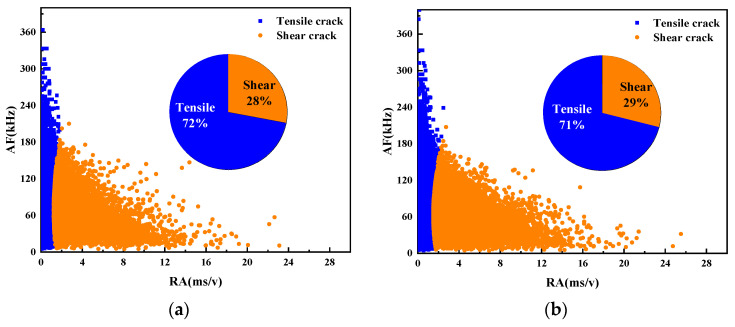
Scatter plots of RA and AF of UHPMC with different stone powder contents: (**a**) 0% stone powder content; (**b**) 10% stone powder content.

The fracture modes of UHPC notch beams with different stone powder contents were compared according to the AE parameters RA and AF. The RA and AF scatter plots of the M60-S0-W0.2 and M60-S10-W0.2 beam specimens with stone powder contents of 0% and 10% are shown in Figure 30.

In the comparison of both beams, it is found that both exhibit a combination of tensile and shear damage, predominantly characterized by tensile cracks. Notably, the M60-S0-W0.2 beam demonstrates a higher AF value but a lower RA value. It is evident that the shear performance of beams with a 0% stone powder content is higher than that of those with a 10% stone powder concentration. This is because the excessive stone powder content in the M60-S10-W0.2 beam weakens the supportive effect of the skeleton, resulting in a sparser internal structure and slightly weakened tensile resistance. However, the difference between the two is not significant.

## 4. Conclusions

This study investigates the mechanical and fracture properties of UHPMC materials with varying levels of MS replacement rate, water–binder ratio, and stone powder content, using a combination of compressive strength tests, four-point bending tests, and AE technology. The following conclusions can be drawn:

(1) The compressive strength, stiffness, and elastic modulus of UHPMC are positively correlated with the MS replacement rate and negatively correlated with the water–binder ratio. Additionally, the compressive strength shows an increasing-then-decreasing trend with increasing stone powder content. When the stone powder content is 5%, the compressive strength shows the best performance.
(2) Increasing the MS replacement ratio or reducing the water–binder ratio enhances the flexural strength, stiffness and modulus of elasticity of UHPMC, while increasing the stone powder content initially increases and then decreases the flexural strength of UHPMC. The coefficient of the influence of the MS replacement ratio on the flexural toughness ratio is 0.031, indicating a slightly positive effect.
(3) UHPC specimens with a 100% MS replacement ratio or a 0.16 water–binder ratio exhibit smaller acoustic emission signals during compression and bending processes, indicating delayed crack initiation and fewer cracks. The *b*-value and entropy fluctuation are more stable. Compared to specimens with a 0% stone powder content, UHPC specimens with a 10% stone powder content show later failure, indicating a stronger resistance.
(4) In the four-point bending test of UHPMC beams, the damage primarily occurs in the form of a tensile–shear mixed failure mode, but tensile failure accounts for more than 70% of the damage. A 0.16 water–binder ratio and a 100% MS replacement ratio can suppress tensile cracks and increase shear cracks. The specimens with 0% and 10% stone powder content have little impact on the crack pattern.

In summary, the MS made by grinding waste rocks has the advantages of easy access, low price and environmental protection. It is a favorable replacement material for the sustainable development of UHPC. Based on this experiment, it is evident that the difficulty in achieving the required performance of MS is mainly due to the high content of stone powder and the uneven gradation. Therefore, in actual production, it is essential to strictly control the proportion of stone powder and to screen the MS particles within the range of 0.15–1.18 mm. However, in current structural construction, the use of MS remains relatively limited. If local aggregates can be fully utilized, the rational utilization of resources and a reduction in costs can be achieved. Furthermore, the types of rocks vary in different regions. It is thus necessary to further study the performance of aggregates in various regions to explore the applicability of MS.

## Figures and Tables

**Table 1:** Aggregate performance index.

Index	Unit	MS	QS
Fineness modulus	/	2.50	2.44
SP content	%	3.2	-
Methylene blue value	MB	0.5	-
Crushing value index	%	17.14	5.28
Apparent density	kg/m^3^	2799	2631
Bulk density	kg/m^3^	1790	1730
Voidage	%	36	34

**Table 2:** UHPMC mix design (kg/m^3^).

Group	Cement	SF	QS	MS	SP	QP	Steel Fiber	Water	HRWRA
M20-S5-W0.16	850	195.5	748	177.65	9.35	331.5	148.75	167.28	42.5
M20-S5-W0.2	850	195.5	748	177.65	9.35	331.5	148.75	209.1	42.5
M20-S0-W0.18	850	195.5	748	187	-	331.5	148.75	188.19	42.5
M20-S10-W0.18	850	195.5	748	168.3	18.7	331.5	148.75	188.19	42.5
M60-S5-W0.18	850	195.5	374	532.95	28.05	331.5	148.75	188.19	42.5
M60-S5-W0.18	850	195.5	374	532.95	28.05	331.5	148.75	188.19	42.5
M60-S10-W0.2	850	195.5	374	504.9	56.1	331.5	148.75	209.1	42.5
M60-S0-W0.16	850	195.5	374	561	-	331.5	148.75	167.28	42.5
M60-S0-W0.2	850	195.5	374	561	-	331.5	148.75	209.1	42.5
M60-S10-W0.16	850	195.5	374	504.9	56.1	331.5	148.75	167.28	42.5
M60-S5-W0.18	850	195.5	374	532.95	28.05	331.5	148.75	188.19	42.5
M60-S5-W0.18	850	195.5	374	532.95	28.05	331.5	148.75	188.19	42.5
M60-S5-W0.18	850	195.5	374	532.95	28.05	331.5	148.75	188.19	42.5
M100-S5-W0.16	850	195.5	-	888.25	46.75	331.5	148.75	167.28	42.5
M100-S5-W0.2	850	195.5	-	888.25	46.75	331.5	148.75	209.1	42.5
M100-S0-W0.18	850	195.5	-	935	-	331.5	148.75	188.19	42.5
M100-S10-W0.18	850	195.5	-	841.5	93.5	331.5	148.75	188.19	42.5

Note: silica fume, quartz sand, manufactured sand, stone powder, quartz powder and high-performance polycarboxylic acid water-reducing additive are abbreviated as SF, QS, MS, SP, QP and HPWRA, respectively. All material units are in kg/m^3^. M“X”-S“Y”-W“Z” denotes reinforced UHPMC specimens with a MS replacement ratio X% (X = 20, 60 and 100), a stone powder content Y% (Y = 0, 5 and 10), and a water–binder ratio Z (Z = 0.16, 0.18 and 0.2).

**Table 3:** Optimal solution of prediction target.

Water–Binder Ratio	Stone Powder Content (%)	MS Replacement Ratio (%)	Compressive Strength (MPa)	Flexural Strength (MPa)	Flexural Toughness Ratio	Confidence
0.162	5.05	100	137.221	14.505	0.436	95%

**Table 4:** Displacement ductility ratio.

Factor	Group	Level	Yield Displacement	Limit Displacement	Displacement Ductility Ratio
Water–binder ratio	M20-S5-W0.16	0.16	1.586	5.019	3.164
	M20-S5-W0.2	0.2	2.059	5.658	2.748
	M60-S0-W0.16	0.16	1.333	5.504	4.128
	M60-S0-W0.2	0.2	1.397	4.608	3.297
	M100-S5-W0.16	0.16	1.598	6.608	4.136
	M100-S5-W0.2	0.2	1.812	5.74	3.168
MS replacement ratio	M20-S5-W0.16	20%	1.586	5.019	3.164
	M100-S5-W0.16	100%	1.598	6.608	4.136
	M20-S5-W0.2	20%	2.059	5.658	2.748
	M100-S5-W0.2	100%	1.812	5.74	3.168
	M20-S0-W0.18	20%	1.549	4.478	2.891
	M100-S0-W0.18	100%	1.217	4.79	3.936
Stone powder content	M20-S0-W0.18	0%	1.549	4.478	2.891
	M20-S10-W0.18	10%	1.617	4.169	2.578
	M60-S0-W0.16	0%	1.333	5.504	4.128
	M60-S10-W0.16	10%	1.809	4.615	2.551
	M100-S0-W0.18	0%	1.217	4.79	3.936
	M100-S10-W0.18	10%	2.958	5.549	1.876

## Data Availability

The data presented in this study are available upon request from the corresponding author.
